# Chronic active Epstein-Barr virus infection with reinfection of SARS-CoV-2: a case report

**DOI:** 10.1186/s12985-024-02418-7

**Published:** 2024-06-24

**Authors:** Hongmei Wu, Li Liu, Jialin Qu, Chunrui Wang, Xiaofeng Shi, Yu Lei

**Affiliations:** 1 Key Laboratory of Molecular Biology for Infectious Diseases (Ministry of Education), Institute for Viral Hepatitis, Department of Infectious Diseases, The Second Affiliated Hospital, Chongqing Medical University, No.288 Tianwen Rd., Nan Ping District, Chongqing, 400060 People’s Republic of China; 2 Department of Pathology, The Second Affiliated Hospital, Chongqing Medical University, No.288 Tianwen Rd., Nan Ping District, Chongqing, 400060 People’s Republic of China

**Keywords:** SARS-CoV-2, Chronic active Epstein Barr virus (CAEBV), Co-infection, Reinfection

## Abstract

We describe the case of a 57-year-old male with jaundice, abdominal distension and fatigue. He was diagnosed as chronic active Epstein-Barr virus infection (CAEBV) due to intermittent elevated liver enzymes, hepatosplenomegaly and pancytopenia, with persistent positive of EBV biomarkers in blood and also positive in liver tissue. The patient was reinfected by SARS-CoV-2 within 2 months companied with CAEBV. The patient’s second infection with SARS-CoV-2 led to the aggravated liver dysfunction with pneumonia and re-admission. After receiving symptomatic treatment, the patient showed significantly improvement of symptoms with partially restoration of liver function. After discharge, the patient’s health status continued to deteriorate and eventually died. The instances of SARS-CoV-2 co-infection with the original chronic virus are not uncommon, but the exact mechanism of EBV and SARS-CoV-2 coinfection and the relationship between them are still unclear. Since co-infection of SARS-CoV-2 with original chronic virus might affect each other and lead disease aggravated and complicated, it is necessary to differentiate in the diagnosis of disease and it is important to be aware of the re-infection signs of SARS-CoV-2 in people with chronic virus infection diseases, as well as the risk of co-infection of SARS-CoV-2 with other viruses.

## Introduction

Epstein-Barr virus (EBV) belongs to the human herpesvirus family and is a ubiquitous virus. It is estimated that more than 90% of the adults worldwide are infected with this virus. In majority cases, EBV infection is asymptomatic while sometimes it could develop to acute infectious mononucleosis syndrome [1]. Chronic EB virus infections are rare but can occur. Chronic active EBV infection (CAEBV) is a high-mortality disease with a high EBV loads in the peripheral blood of patients [2]. It is characterized by notablely hepatosplenomegaly, lymphadenopathy, fever, frequent peripheral blood cytopenias, and polyclonal hypergammaglobulinemia [3]. The course of disease may wax and wane or be fulminant [4]. Numerous life-threatening complications can occur in patients with CAEBV, including malignant lymphoma, transplantation-related complications, hepatic failure, hemophagocytic syndrome, digestive tract bleeding/perforation, leukemia, multiple organ failure, and some unknown reasons [5].

Coronavirus disease 2019 (COVID-19) is caused by severe acute respiratory syndrome coronavirus 2 (SARS-CoV-2) [6] and arouse a worldwide pandemic. The number of infection and re-infection cases continues to increase. As of May 2024, over 775 million confirmed cases and over 7.0 million deaths have been reported worldwide [7]. Chronic liver disease, diabetes, hypertension, older age and obesity are common risk factors for severe COVID-19 [8]. The latest data from various countries suggest that the rates of re-infection ranging from 5 to 15%, although this proportion is expected to increase as time passes [9]. However, the mechanism and clinical manifestations of co-infection of EBV with SARS-CoV-2 are still not clear. Herein, we report a case of the CAEBV patient who was twice infected with SARS-CoV-2 within 2 months.

## Case presentation

In July 2022, a 57-year-old male presented with jaundice for 10 days. He complained of yellowing skin and sclera for 10 days, accompanied with fatigue, poor appetite, abdominal distension and pain for 3 months. In April 2022, he was hospitalized in another hospital for abdominal distension, abdominal pain, and recurrent fever (maximum temperature 39.7 °C). After completing a series of tests (Specific information not available), he was diagnosed with suspected tuberculous peritonitis and had received diagnostic anti-tuberculosis treatment with isoniazid, rifampicin and ethambutol for 1 month. The bone marrow biopsy and hepatitis virus markers were negative. He had a 2-year history of thrombocytopenia and unexplained intermittent hepatic dysfunction.

On admission, he showed uncomfortable appearance. Physical examination revealed his body temperature was 36.5 °C, blood pressure 110/80 mmHg, pulse 78 beats per minute, respiratory rate 18 breaths per minute and oxygen saturation 98%. His skin and sclera were obviously jaundiced. Cardiopulmonary examination revealed no abnormalities. The abdomen was distended with high tension. An enlarged spleen was palpable 3 cm below the left rib.

Laboratory findings on admission were as shown in Table 1: thrombocytopenia (platelets of 48 × 10^9/L), leukopenia (white blood cells 1.56 × 10^9/L with 70.9% neutrophils) and mild anemia (hemoglobin of 121 g/L). Liver function tests showed elevated serum bilirubin levels, total bilirubin (TB) 80.6umol/L, direct bilirubin (DB) 72.7umol/L); elevated alanine aminotransferase (ALT 179 U/L) and aspartate aminotransferase (AST 220 U/L); elevated alkaline phosphatase (ALP 390 U/L) and gamma-glutamyl transferase (GGT 395 U/L). Meanwhile, EB IgG level increased notably in the plasma. EB virus DNA was positive in both plasma and lymphocytes. Further laboratory results of the patient are shown in Table 1. The patient’s bone marrow pathology suggested approximately normal myeloproliferation; bone marrow cytology showed delayed maturation of the granulocyte lineage and blocked maturation of megakaryocytes. Autoantibodies for autoimmune liver disease were negative except for mildly elevated anti-mitochondrial antibody M2 (AMA-M2) (30.1RU/ml). Further laboratory results of the patient are shown in Table 2 and Table 3.

**Table 1:** Laboratory indices of the patient at admission and after treatment

**Laboratory test**	**First hospital admission**		**Second hospital admission**		**Normal range**
	Admission result	Post-treatment Results	Admission result	Post-treatment Results	
**LFT**					
AST	220	34	72	26	15–40 U/L
ALT	179	25	55	22	9–50 U/L
TB	80.6	30.2	80.2	71.6	5.1–28.0 umol/L
DB	72.7	25.3	74.4	64.7	0.0–10.0 umol/L
ALP	390	309	271	225	45–125 U/L
GGT	395	282	323	276	10–60 U/L
**CBC**					
Hb	121	103	112	119	130–175 g/L
WBC	1.56	2.23	1.83	2.25	(3.50–9.50)*10^9/L
RBC	3.96	3.39	3.58	3.86	(4.30–5.80)*10^9/L
PLT	48	64	52	55	(100–300) × 10^9/L
LYM	0.32	0.62	0.54	0.76	(1.10–3.20)*10^9/L
**Renal function**					
BUN	5.89	3.34	5.63	4.69	3.10–8.00 mmol/L
Cr	71.2	53.9	43.6	41.8	57.0–97.0 mmol/L
**Coagulation**					
PT	12.4	12.6	12.9	13.2	11.0–14.5
INR	0.91	0.93	0.96	0.99	0.70–1.30
**Cytokines**					
IL-2R	1225.00	**-**	**-**	**-**	223.00–710.00U/ml
IL-6	16.4	**-**	**-**	**-**	0–5.90 pg/ml
IL-8	152.00	**-**	**-**	**-**	0–62.00 pg/ml
TNF-α	18.4	**-**	**-**	**-**	0.00–8.10 pg/ml
**PCT**	0.16	0.16	0.14	0.21	0–0.05 ng/ml
**ESR**	17				0-15 mm/h
**CA125**	57.7				0-35U/ml
**CRP**	< 5	< 5	5.15	< 5	< 10 mg/L
**AFP**	4.73			4.30	0–13.2ug/L
**Immunoglobulin**					
IgG	14.7				7.51–15.6 g/L
IgA	1.59				0.82–4.53 g/L
IgM	0.75				0.46–3.04 g/L
**COVID-19 Test(PT-PCR)**					
**ORF1ab gene**	**Negative**	**-**	**Positive (CT:36.1)**	**-**	negative
**N gene**	**Negative**	**-**	**Positive (CT:34.5)**	**-**	negative
**COVID-19 antigen**	**Negative**	**-**	**Positive**		negative
**EBV-DNA(Plasma)**	2.43 × 10^4	8.0 × 10^4	5.35 × 10^4	**-**	< 1 × 10^3 coples/ml
**EBV-DNA(Lymphocytes)**	5.9 × 10^6	8.5 × 10^5	1.88 × 10^5	**-**	< 1 × 10^3 coples/ml
**EBV nuclear antibody IgG**	112	85.10	103.00	**-**	5.00–20.00U/ml
**EBV capsid antibody IgG**	> 750	> 750	> 750	**-**	0.00–20.00U/ml
**EBV early antibody IgG**	> 150	> 150	> 150	**-**	0.00–40.00U/ml
**EBV capsid antibody IgM**	< 10.0	< 10.0	< 10.0	**-**	0.00–40.00U/ml

*CBC* Complete blood count, *LFT* Liver function test, *RBC* Red blood cell, *Hb* Hemoglobin, *HCT* Hematocrit, *WBC* White blood cell, *PLT* Platelets, *NEU%* Granulocyte, *LY%* Lymphocyte, *ALB* Albumin, *ALT* Alanine aminotransferase, *AST* Aspartate aminotransferase, *T-BIL* Total bilirubin, *D-BIL* Direct bilirubin, *ChE* Cholinesterase, *γ-GT* Glutamyl transferase, *ALP* Alkaline phosphatase, *BUN* Blood urea nitrogen, *Cre* Creatinine, *GFR* Glomerular filtration rate, *K* Kalium, *Na* Natrium, *Cl* Chloride, *PT* Prothrombin time, *PTA* Prothrombin activity, *INR* International normalized ratio, *PCT* Procalcitonin, *ESR* Erythrocyte sedimentation rate, *CRP* C-reactive protein, *AFP* Alpha-fetoprotein, *IL-2R* Interleukins-2R, *TNF-α* Tumor necrosis factor-α, - no detection

**Table 2:** The rest of the patient’s test results

**Laboratory test**	**Results**	**Reexamination**
Hepatophilic virus		
HAV	negative	
HBV		
HCV		
HEV		
AMA-M2	30.1RU/ml	15.9 RU/ml
HIV	negative	
Mycobacterium tuberculosis(IgG and IgM)	positive	
Tuberculin pure protein derivative (PPD) test	negative	
And tuberculosis T-cell spot (T-spot) test	negative

**Table 3:** The data regarding peripheral lymphocyte subsets

Name	Results	Reference value
B cell percentage CD5^+^CD3^−^CD19^+^	2.09%	6.48–16.64
NKT cell percentage CD45^+^CD3^+^CD56^+^	1.74%	3.00–8.00
NK cell percentage CD45^+^CD3^−^CD56^+^	30.5%	5.17–24.65
T4 cell percentage CD45^+^ CD3^+^CD4^+^ (t4xbbfl)	50.91%	24.93–45.57
T8 cell percentage CD45^+^ CD3^+^CD8^+^ (t8xbbfl)	19.37%	16.4–33.76
T4 cell count CD45^+^ CD3^+^CD4^+^	0.32*10^9^	(0.20–1.82) *10^9^
T8 cell count CD45^+^ CD3^+^CD8^+^	0.12*10^9^	(0.13–1.35) *10^9^

**Fig. 1:**
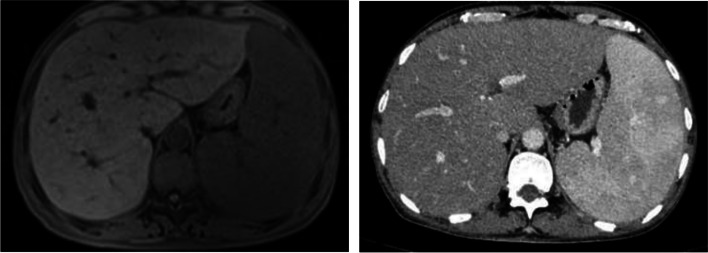
Contrast-enhanced MRI + DWI and Contrast-enhanced CT scan of the abdomen. Left panel: Abdominal magnetic resonance imaging (MRI) at July 2022; Right panel: Abdominal contrast enhanced CT scan at February 2023

**Fig. 2:**
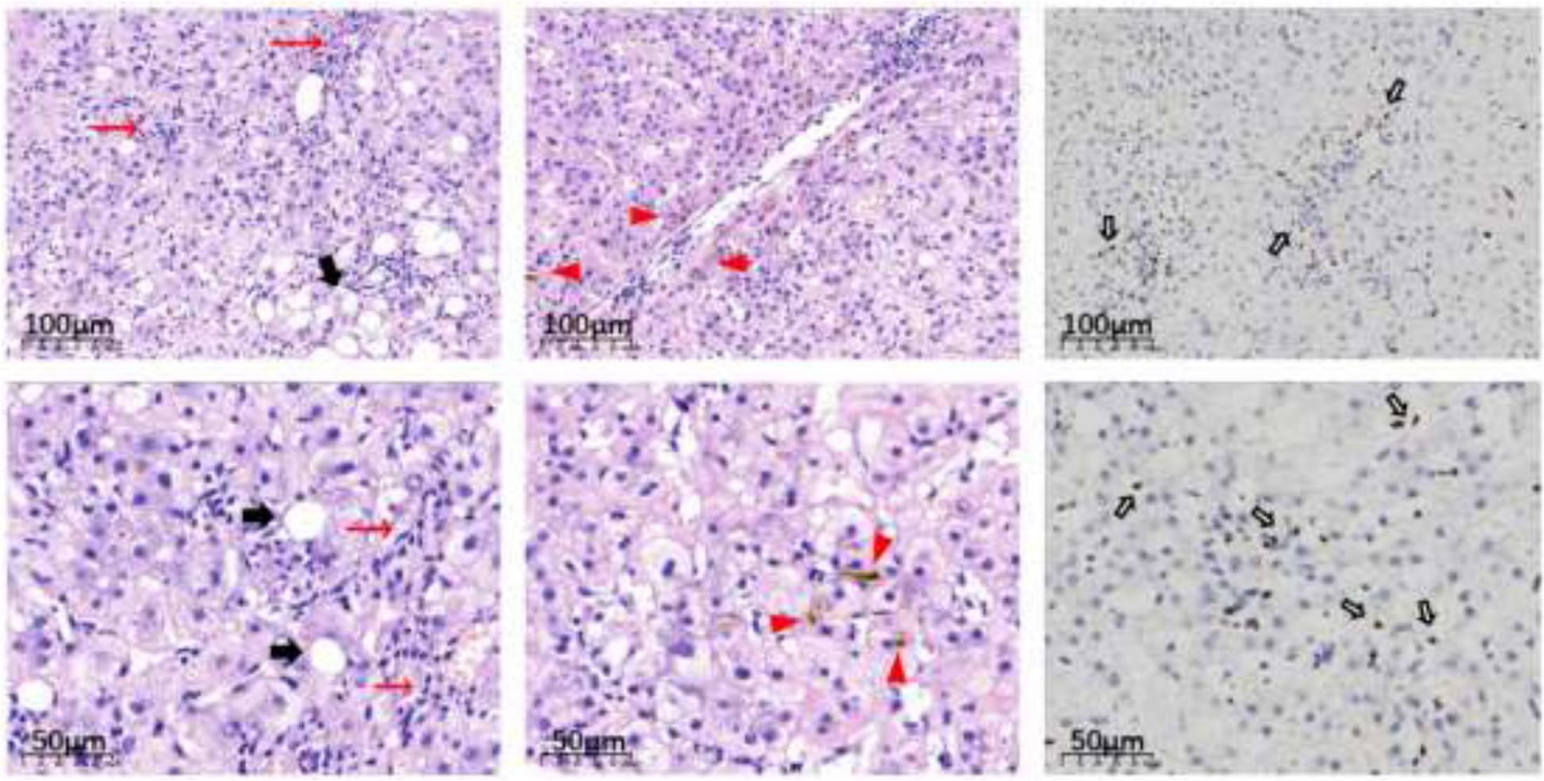
Histology of liver. Left and middle panel: Hematoxylin-eosin (HE) staining of the liver tissue. Right panel: Epstein-Barr virus-encoded small RNAs in situ hybridization (EBER-ISH) of the liver tissue. Scale bars: 100 μm in upper panel and 50 μm in lower panel. The lesion is marked by: → Portal area and lobular inflammation,  hepatocellular and canalicular cholestasis,  steatosis of hepatocytes,  EBER-ISH positive lymphocytes

Contrast-enhanced diffusion weighted magnetic resonance imaging (MRI DWI) of the abdomen showed hepatosplenomegaly (Fig. 1 left panel). Liver biopsy was performed on the patient 1 week after admission. Liver pathology showed total lobular inflammation, moderate portal inflammation, hepatocellular and canalicular cholestasis. The lymphocytes in the sinuses were arranged in a string of beads. The portal bile ducts were normality without inflammation and detachment. EBER-ISH staining of lymphocytes in both lobular and portal was positive (in Fig. 2). All imaging examinations did not indicate lymph node enlargement.

Half a month after admission, the patient developed a high fever, with a maximum temperature 39℃. The patient had elevated infection indicators and a chest CT scan revealed bilateral pneumonia, but none of the blood cultures were abnormal. After successive administration of moxifloxacin, piperacillin tazobactam and bisopenem to fight infection, the patient’s temperature dropped to normal. The patient was diagnosed with EBV hepatitis based on the medical history and examination results and treated with adenosylmethionine, compound glycyrrhizin, ursodeoxycholic acid and ganciclovir for 2 weeks. Liver function recovered (Table 1), while EBV-DNA did not decrease significantly until discharge (August 29, 2022).

In December 2022, 4 months after discharge, coincidentally during the COVID-19 pandemic in China, the patient was found to have a low fever by telephone follow-up and diagnosed with COVID-19 by nucleic acid testing in local hospital. He had sore throat, stuffy nose, and low fever of 37.6℃. Due to the patient’s mild symptoms, he did not go to the hospital. A week later, all symptoms disappeared and SARS-co-2 nucleic acid turned negative. Telephone follow-up also revealed that the patient had received three doses of COVID-19 vaccine 1 year before the onset of symptoms.

**Fig. 3:**
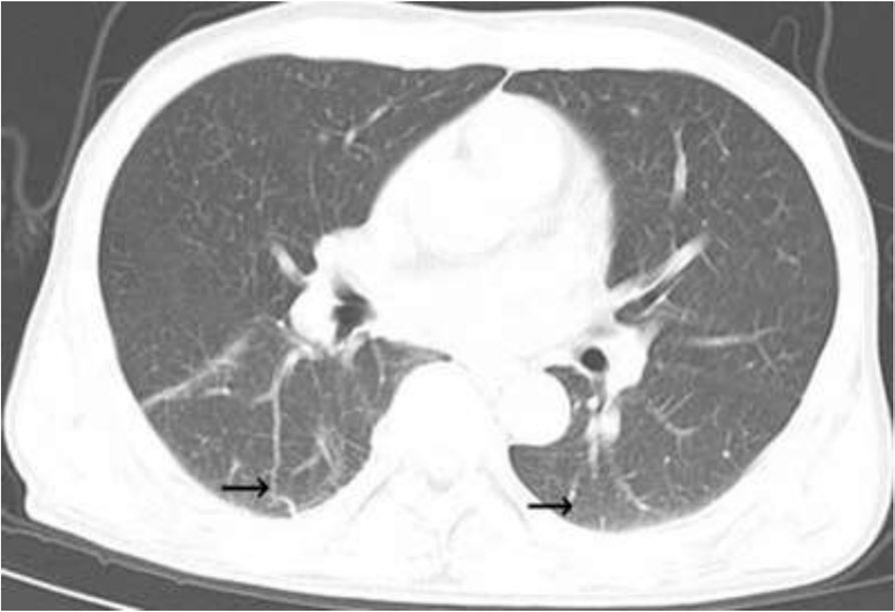
Lung CT scan. Lung CT scan imaging at February 2023. → signs of pulmonary diffuse inflammation

In February 2023, which was 6 months after discharge from our hospital, the patient was readmitted because of obvious jaundice and skin pruritus. During the hospitalization, the patient developed cough and fever. Physical examination revealed that his body temperature was 37.8 °C, blood pressure 106/77 mmHg, pulse rate 84 beats per minute, respiratory rate 20 breaths per minute and oxygen saturation 97%. Laboratory test results on admission remained notable for thrombocytopenia, leukopenia, mild anemia (Table 1). Liver function tests showed elevated serum bilirubin levels, and ALT, AST, ALP and GGT were again elevated (Table 1). EBV DNA, EBV IgM and EBV IgG were still positive. COVID-19 Test [real time Polymerase Chain Reaction (RT-PCR)] of nasopharyngeal swab was positive (ORF1ab gene CT value: 36.1, N gene CT value: 34.5). Chest CT scan revealed bilateral scattered pneumonia (Fig. 3). The patient was diagnosed with CAEBV and co-infected with SARS-CoV-2. The patient received oxygen inhalation but not antiviral therapy since the CT value of COVID-19 Test was not low. At the same time, he also received compound glycyrrhizin and adenosylmethionine for liver dysfunction and jaundice. During the course of the disease, the patient had persistent low fever and was empirically treated with 4.5 g of piperacillin tazobactam per 8 h a day for one week. After 10 days of treatment, the patient was discharged with symptomatic improvement and partial recovery of liver function. The last hospitalization was in May 2023, when the patient presented with yellowing of the skin and sclera, asthenia and abdominal distension. Liver function tests demonstrated severe Liver Impairment. EBV was positive in peripheral blood and lymphocytes. Though the patient was treated aggressively with symptomatic therapy and liver function review showed liver enzymes decreased notably, there was still no remarkable improvement in symptoms. The patient was therefore discharged from the hospital after abandoning treatment. Telephone follow-up revealed that the patient died approximately 1 month after abandoning treatment.

## Discussion

The clinical manifestations of EBV infection are diverse, including fever, persistent lymphadenopathy, splenomegaly, pancytopenia, pruritus and diarrhea. However, the reason for the variability of symptoms in normal hosts of varying ages in still unknown. CAEBV is a rare but high-mortality, high-morbidity disease with life-threatening complications [10]. Although the pathological mechanism was still unclear, recent studies have indicated that clonal expansion of EBV infected T cells and NK cells plays a central role in the pathogenesis of CAEBV [11]. In our case, the patient was persistently positive for EBV for more than half a year or longer, accompanied with hepatosplenomegaly, persistent peripheral blood cytopenias, frequent liver function damage. The patient has been diagnosed with tuberculous peritonitis at another hospital and received antituberculosis drugs for 1 month. However, we did not find any evidence of active tuberculosis infection, PPD test、T-spot test were negative. The repeated bone marrow biopsy was negative for leukemia, lymphoma, and myelodysplastic syndrome. Liver function damage in this patient was mainly manifested by cholestasis, with significantly elevation of TBIL, AKP and GGT (Table 1). Combining with the medication history, the patient was susceptible to misdiagnosis of drug-induced liver injury (DILI), although DILI could not be ruled out since that antituberculotic drugs might have a side effect on liver function. On the other hand, this patient had a positive AMA-M2, which could be cholestasis due to primary biliary cholangitis. However, liver pathology showed typical hepatitis caused by EBV rather than intrahepatic bile duct damage (Fig. 2). The EBV is also positive in lymphocytes of peripheral blood and liver tissue. It could be confirmed that the patient’s liver function damage was caused by EB virus infection. Hemophagocytic lymphohistiocytosis (HLH) is a fatal inflammatory syndrome characterized by excessive activation of immune cells. According to the diagnostic criteria of HLH, the patient did not meet at least five of the eight diagnostic criteria. The patient had symptoms such as “fever and splenomegaly” with cytopenias affecting at least two of three lineages in the peripheral blood and ferritin > 500ug/ml, but no hemophagocytosis on bone marrow biopsy, no hypertriglyceridemia and/or hypofibrinogenemia, and no obvious abnormalities of the natural killer (NK) cells and soluble IL-2 receptor less than 2400 U/mL [12], so the presence of EBV-related hemophagocytic syndrome was not considered.

Treatment of chronic active EBV infection was difficult. Antiviral or immunomodulatory agents, such as acyclovir, ganciclovir, vidarabine, interferon-a, and interleukin-2 (IL-2), have been tested with limited effection in patients with CAEBV. And monoclonal antibodies—Rituximab also inhibits EBV replication [13]. The indications for the use of rituximab in China are non-Hodgkin’s lymphoma(NHL), chronic lymphocytic leukemia(CLL), rheumatoid arthritis(RA) and vasculitis. However, the patient did not have an indication for appeal. Medical workers must strictly follow the indications of the drug, so the patient was not treated with rituximab. Ganciclovir is a commonly used antiviral medication that is clinically used to treat the herpes virus. Ganciclovir inhibits the binding of deoxyguanosine trivalent phosphate to DNA polymerase, resulting in the cessation of DNA prolongation, thereby preventing DNA virus replication [14], EBV is a DNA virus. In this case, we used ganciclovir for EBV infection at the first admission. However, it has limited effect on this patient since the EBV-DNA loading didn’t decrease after therapy. We also used adenosylmethionine and glycyrrhizin. Both have hepatoprotective effects, significantly lowering serum aminotransferases, reducing inflammation levels and improving liver histology, and the latter also has antiviral effects: causing growth restriction and irreversible loss of infectivity of herpes simplex virus [15]. And liver function has been restored for a while.

From December 2022, China has announced to optimize COVID-19 control strategies. The continuous spread of Omicron variants persisted in China, reaching more provinces and more local areas throughout 2022 [16]. It was estimated that at least 60–80% of people in major cities were infected in this major outbreak [17]. This patient was infected with COVID-19 twice in a short period of time, leading to a second admission to the hospital and a diagnosis of EBV/SARS-CoV-2 co-infection. Therefore, it is considered that persistent EBV infection may cause re-infection with SARS-CoV-2 in a short period of time, but further validation is needed. Several researches have shown that SARS-CoV-2 infection decreases human cellular immunity [18, 19], and might activate latent EBV in patients. Dinesh Verma, et al. stated that EBV infection affects the efficiency and severity of SARS-CoV-2 infection by increasing the expression of ACE2, the cellular receptor for SARS-CoV-2, on epithelial cells [20]. Six EBV/SARS-CoV-2 co-infection cases [11, 21–25] have so far been reported globally. These cases however did not particularly suggest any increased risk of COVID-19 in EBV-infected patients. By reviewing these case reports and a retrospective single-center study [16], it was shown that EBV/SARS-CoV-2 co-infection exacerbates patients’ symptoms. EBV reactivation may be associated with the severity of COVID-19. The clinical features of EBV/SARS-CoV-2 co-infection were variable and complex, which also might lead to a missed diagnosis.

It was difficult to distinguish which virus caused the fever, aggravated liver disfunction and pneumonia of this case in the second admission, which actually might be the consequences of EBV/SARS-CoV-2 co-infection. The treatment of EBV/SARS-CoV-2 co-infection is timely antiviral therapy and management of complications. However, we just took symptomatic treatment instead of antiviral treatment in the second admission of this case. Before discharge, the patient’s symptoms improved significantly with partially recovered liver function. In the normal population, SARS-CoV-2 infection occurring almost 6 months after initial infection is considered as re-infection [26, 27]. However, in this case, the interval between twice infections of SARS-CoV-2 was within 60 days, which was markedly shorter than that in the normal population. Persistent EBV infection has been proposed as a possible driver of SARS-CoV-2 reinfection within a short period of time [16].

To sum up, the patient’s diagnosis needs to consider several diseases or the overlap of several diseases. The cause of the patient’s death is still unknown, perhaps liver failure, but it is uncertain whether lymphoma or other conditions occurred.

The limitation of this case is that we didn’t examine variants of SARS-CoV-2 in the two infections in this patient, while it is still difficult to distinguish viral re-infection from persistent or long-term infections [28]. However, our research highlights the importance of detecting co-infection with other viruses in certain cases of confirmed EBV infection or SARS-CoV-2, and vice versa.

## Conclusion

In conclusion, we describe a 57-year-old patient with CAEBV infection combined with SARS-CoV-2 re-infection. Co-infection of these two viruses lead to the complicated and exacerbated clinical manifestations. EBV may shorten the interval between SARS-CoV-2 reinfections, while SARS-CoV-2 infection might lead to EBV reactivation. Clinicians should be aware of the risk of the signs of SARS-CoV-2 re-infection in people with chronic viral infectious diseases, as well as the risk of co-infection of SARS-CoV-2 with other viruses, especially during pandemics.

## Data Availability

No datasets were generated or analysed during the current study.

## Abbreviations

COVID-19: Coronavirus disease 2019
SARS-CoV-2: Severe acute respiratory syndrome coronavirus 2
CBC: Complete blood count
RBC: Red blood cell
Hb: Hemoglobin
HCT: Hematocrit
WBC: White blood cell
PLT: Platelets
NEU: Granulocyte
LY: Lymphocyte
LFT: Liver function test
ALB: Albumin
ALT: Alanine aminotransferase
ALP: Alkaline phosphatase
AST: Aspartate aminotransferase
T-BIL: Total bilirubin
D-BIL: Direct bilirubin
ChE: Cholinesterase
GGT: Gamma-glutamyl transferase
BUN: Blood urea nitrogen
Cre: Creatinine
GFR: Glomerular filtration rate
K: Kalium
Na: Natrium
Cl: Chloride
PT: Prothrombin time
PTA: Prothrombin activity
INR: International normalized ratio
PCT: Procalcitonin
ESR: Erythrocyte sedimentation rate
CRP: C-reactive protein
AFP: Alpha-fetoprotein
IL-2R: Interleukins-2R
TNF-α: Tumor necrosis factor-α
CA125: Carbohydrate antigen-125
AMA-M2: Anti-mitochondrial antibody M2
IgG: Immunoglobulin G
IgM: Immunoglobulin M
IgA: Immunoglobulin A
HAV: Hepatitis A Virus
HBV: Hepatitis B Virus
HCV: Hepatitis C Virus
HEV: Hepatitis E Virus
HIV: Human lmmunodeficiency Virus
PPD: Purified Protein Derivative
T-SPOT: T-cell spot of tuberculosis assay
DILI: Drug-induced liver injury
IL-2: Interleukin-2
RT-PCR: Real time Polymerase Chain Reaction
